# A novel *Saclayvirus Acinetobacter baumannii* phage genomic analysis and effectiveness in preventing pneumonia

**DOI:** 10.1007/s00253-024-13208-0

**Published:** 2024-07-27

**Authors:** Shibin Li, Bingdong Wei, Le Xu, Cong Cong, Bilal Murtaza, Lili Wang, Xiaoyu Li, Jibin Li, Mu Xu, Jiajun Yin, Yongping Xu

**Affiliations:** 1 School of Bioengineering, Dalian University of Technology, Dalian, 116024 China; 2 Institute of Animal Nutrition and Feed Science, Jilin Academy of Agricultural Sciences, Gongzhuling, 136100 China; 3 Liaoning Innovation Center for Phage Application Professional Technology, Dalian, 116620 Liaoning China; 4 Dalian SEM Bio-Engineering Technology Co. Ltd., Dalian, 116620 China; 5 Department of General Surgery, Affiliated Zhongshan Hospital of Dalian University, Dalian, 116300 China

**Keywords:** Bacteriophage, *Acinetobacter baumannii*, Multi-drug resistant, Neutrophil-deficient, Pneumonia prophylactic

## Abstract

**Abstract:**

*Acinetobacter baumannii*, which is resistant to multiple drugs, is an opportunistic pathogen responsible for severe nosocomial infections. With no antibiotics available, phages have obtained clinical attention. However, since immunocompromised patients are often susceptible to infection, the appropriate timing of administration is particularly important. During this research, we obtained a lytic phage vB_AbaM_P1 that specifically targets *A. baumannii*. We then assessed its potential as a prophylactic treatment for lung infections caused by clinical strains. The virus experiences a period of inactivity lasting 30 min and produces approximately 788 particles during an outbreak. Transmission electron microscopy shows that vB_AbaM_P1 was similar to the *Saclayvirus*. Based on the analysis of high-throughput sequencing and bioinformatics, vB_AbaM_P1 consists of 107537 bases with a G + C content of 37.68%. It contains a total of 177 open reading frames and 14 tRNAs. No antibiotic genes were detected. In vivo experiments, using a cyclophosphamide-induced neutrophil deficiency model, tested the protective effect of phage on neutrophil-deficient rats by prophylactic application of phage. The use of phages resulted in a decrease in rat mortality caused by *A. baumannii* and a reduction in the bacterial burden in the lungs. Histologic examination of lung tissue revealed a decrease in the presence of immune cells. The presence of phage vB_AbaM_P1 had a notable impact on preventing *A. baumannii* infection, as evidenced by the decrease in oxidative stress in lung tissue and cytokine levels in serum. Our research offers more robust evidence for the early utilization of bacteriophages to mitigate *A. baumannii* infection.

**Key points:**

•*A novel Saclayvirus phage infecting A. baumannii was isolated from sewage.*

•*The whole genome was determined, analyzed, and compared to other phages.*

•*Assaying the effect of phage in preventing infection in neutrophil-deficient models.*

**Supplementary Information:**

The online version contains supplementary material available at 10.1007/s00253-024-13208-0.

## Introduction

The emergence of drug-resistant microorganisms poses a significant worldwide issue in public health (Pendleton et al. 2013; WHO 2014), particularly the ESKAPE (*Enterococcus faecalis*, *Staphylococcus aureus*, *Klebsiella pneumoniae*, *Acinetobacter baumannii*, *Pseudomonas aeruginosa*, and *Enterobacter* species) pathogens (Rice 2008). The opportunistic pathogen *Acinetobacter baumannii* is one of the most important hospital-acquired infections (Almasaudi 2018). On a global scale, the level of multi-drug resistance in *A. baumannii* surpasses that of other Gram-negative pathogens. Approximately 44% of *A. baumannii* clinical isolates exhibit resistance to multiple drugs (Giammanco et al. 2017). This particular bacterium is associated with pneumonia, sepsis, infections in wounds, urinary tract infections, and various other ailments (Nie et al. 2020). Immunocompromised patients in intensive care units (ICU) are at great risk due to the emergence of multidrug-resistant bacteria, specifically carbapenem-resistant *A. baumannii* (Wong et al. 2017). In 2013, the urgent need for new antibiotics was emphasized by the Centers for Disease Control and Prevention (CDC) when they categorized *A. baumannii*, which is resistant to carbapenem, as a pathogen with multi-drug resistance (MDR) (Sievert et al. 2013). In 2017, the World Health Organization (WHO) published a roster, highlighting the primary perilous antibiotic-resistant pathogens to worldwide public health, with *A. baumannii* securing the top position (Willyard 2017). In this context, there is a crucial need for novel antimicrobial agents that can overcome carbapenem-resistant *A. baumannii* infections.

An effective approach to address this issue is to use bacteriophages as an alternative therapy strategy that has received growing attention recently (Pires et al. 2015). Phages, which are viruses that specifically target bacteria, are the most prevalent life forms on the planet (Bujak et al. 2020; Dunne et al. 2018). Phages are categorized as either lytic or lysogenic based on their infection life cycle (Pasechnek et al. 2020). Lytic phages invade bacterial hosts and break down the hosts, thereby releasing their progeny. They can serve as a safe and effective alternative to combat multidrug-resistant pathogens (Yuan et al. 2019). Phages have many advantages over conventional antibiotic treatments, particularly their specificity, since they usually target only a single bacterial species and do not interfere with the body’s natural microbiota. Furthermore, the replication of phages relies on the existence of the host, making them self-limiting (Luscher et al. 2020). Studies conducted in vitro and in vivo have demonstrated that phage treatments are superior to antibiotic treatments in terms of precision while also avoiding any negative reactions or tissue toxicity (Barros et al. 2019). Phages have attracted more attention recently in combating infections due to the high bioavailability of infected sites and their ability to replicate and migrate autonomously.

In previous studies, in vivo phage therapies for multidrug-resistant bacterial infections mainly focused on infection treatment (Guillon et al. 2021; Jeon et al. 2016). After infection, phage therapies were quickly administered and showed remarkable results (Palser et al. 2019; Prazak et al. 2019). However, the successful control of any bacteria largely depends on the timing of therapies (Wang et al. 2016), which dramatically limits the flexibility of drug administration. However, because immunocompromised patients are a susceptible population, infection in one patient can rapidly cause infection in other patients in the same unit, making it difficult to determine the timing of infection and administer phage therapy promptly. In such cases, prophylactic use of phage can stop *A. baumannii* infections in the unit.

In our study, we obtained the phage vB_AbaM_P1 of *A. baumannii* from medical sewage and used cyclophosphamide (CTX) to make neutrophil-deficient models (Manepalli et al. 2013). The potential of phage vB_AbaM_P1 for treatment of *A. baumannii* infections was evaluated by physiological characteristics and whole-genome analysis. The preventive effect of vB_AbaM_P1 on pulmonary infections caused by multidrug-resistant *A. baumannii* was studied by prophylactic application using phages under neutrophil-deficient conditions.

## Materials and methods

### Bacterial strains and antimicrobial resistance

The *A. baumannii* utilized in our research was obtained from the respiratory secretions of individuals diagnosed with pneumonia. Identification of collected biological samples was done using VITEK®^2^ Compact. The disk diffusion method is employed to test the susceptibility of clinical isolates to antimicrobial agents by following the guidelines set by the Association of Clinical and Laboratory Standards Institute (CLSI) (Jeon and Yong 2019).

### Animal maintenance

Male Wistar rats, aged six weeks and weighing 180–200 g, were acquired from Liaoning Changsheng Biotechnology Co., Ltd. Throughout the entire experiment, rats were placed in specific pathogen-free (SPF), light-controlled, and temperature-controlled conditions and supplemented with standard feed and water. This study followed the ARRIVE guidelines (https://www.nc3rs.org.uk/arrive-guidelines). Approval for all animal experiments was granted by the Biomedical Ethics Committee of the Dalian University of Technology (Dalian, China; Approval No. DUTSBE220624_02).

### Isolation and purification of phage

Phages were screened and replicated using *A. baumannii* strain CGMCC 1.90331 as a host. The bacteriophage vB_AbaM_P1 was obtained from sewage in a hospital by making some adjustments to a method that had been previously researched (Yuan et al. 2021). Briefly, impurities and bacteria were removed by centrifugation (10,000 × g, 10 min, 4 °C) and filtration (0.22 μm filtration membrane). One hundred milliliters of treated wastewater and 1 mL of *A. baumannii* strain CGMCC 1.90331 in the mid-log phase were combined with 100 mL of 2 × LB broth. To enhance the phages, the mixture was incubated for 12 h at 37 °C and 160 rpm. At a temperature of 4 °C, the culture underwent centrifugation at 10,000 × g for 10 min. Subsequently, the 0.22-μm filter membranes were used to separate the supernatant from the mixture. The phages were detected and purified using the double-layer agar method. A mixture of 100 μL filtrate and 200 μL *A. baumannii* strain CGMCC 1.90331 (mid-log phase) was combined with a semisolid agar medium. The resulting mixture was poured onto LB solid agar plates and incubated at 37 °C for 12 h. Afterward, a transparent phage single plaque was selected and resuspended in 100 μL SM buffer (containing 100 mM NaCl, 10 mM MgSO4, 50 mM Tris–HCl, and 0.01% gelatin). Subsequently, the phage suspension was plated using the double-layer agar technique. To achieve purified phages with consistent plaque appearance, a minimum of three successive individual plaque isolations were carried out. The phage lysates were stored at a temperature of 4 °C. The phage is deposited in the Animal Biotechnology and Nutrition Laboratory, Dalian University of Technology, and is available from the corresponding author.

### High-titer bacteriophage suspension preparation

Phages were concentrated with polyethylene glycol 8000 (PEG8000). Briefly, a final concentration of 1 M NaCl was introduced to 200 mL of phage lysate and kept at a temperature of 4 °C for 6 h. Following this, the phage lysate underwent centrifugation at 10,000 × g for 10 min at a temperature of 4 °C, resulting in the collection of the supernatant. To precipitate phage particles, the supernatant was supplemented with PEG8000 at a final concentration of 10% (w/v). Following an 8-h immersion in ice, phages underwent centrifugation at 4 °C with a force of 10,000 × g for 10 min. Subsequently, they were gathered, precipitated, and reconstituted in 1 mL of SM buffer. The process of purifying concentrated phage suspensions was carried out using CsCl density gradient centrifugation, following the previously described method (Tang et al. 2017).

### Host range

The sensitivity of bacterial strains to the phage was tested using the double agar layer technique and efficiency of plating (EOP) to determine the host range (Islam et al. 2020). Briefly, the phage suspension was diluted continuously, placed on the original or target host bacteria, and incubated at 37 °C for 12 h to see if there was a clear cleavage zone. Plaque clarity was categorized into three groups: clear and highly susceptible (+ +), translucent and partially susceptible ( +), and no plaque (-). The calculation of EOP involved determining the ratio between the phage titer (PFU/mL) in each isolate and the phage titer in the reproductive host.

### Phage morphology

The copper grid, covered in carbon, was coated with a phage suspension containing approximately 10^12^ pfu/ml. Afterwards, the grid was negatively stained using uranyl acetate [2% (w/v), pH 4.0]. Subsequently, this grid was observed under a transmission electron microscope (JEM-2000EX, JEOL, Tokyo, Japan).

### Phage adsorption and one‑step growth curve

Mid-log phase culture of the host bacteria were mixed with vB_AbaM_P1 at an MOI of 0.1. Aliquots of the mixture (100 µl) were collected every 3 min and centrifuged at 10,000 × g, 4 °C, for 5 min. The titer of unadsorbed phages in the supernatant was determined using the double-layer plate method. One-step growth curves were performed as previously described (Wang et al. 2021). Phages were introduced into the mid-log phase culture of the host bacteria at a multiplicity of infection (MOI) of 0.1 and adsorbed at 37 °C for 5 min. After being subjected to centrifugation at a force of 10,000 × g for 10 min, the resulting sediment was reconstituted in a new batch of LB broth. The suspension was agitated at a temperature of 37 °C, and the samples were gathered every 10 min over 90 min. The phage concentrations at each time point were assessed using the double-layer agar technique. All tests were done in triplicate.

### Activity of phage against *A. baumannii*

To assess the antibacterial effectiveness of phages against *A. baumannii*, 200 μL mid-log phase *A. baumannii* strain CGMCC 1.90331 bacterial suspensions were connected to a 96-well plate, and 20 μL of phages was added to the bacterial suspension with different MOIs. Equal amounts of PBS were added to the blank control. All cultures were at 37 °C and 160 rpm, and the OD_600_ was measured every 1 h using a microplate reader for 12 h. All tests were done in triplicate.

### Sequencing analysis

The viral genome extraction kit (Tiangen Biotech, Beijing, China) was utilized to extract the genomic DNA of vB_AbaM_P1. The whole genome of vB_AbaM_P1 was sequenced by Illumina Hiseq 2500 at Sangon Biotech (Shanghai, China). Using PhageTerm to predict phage packaging mechanisms and ends from sequencing results (Garneau et al. 2017). The vB_AbaM_P1 genes were putatively identified by RASTtk (https://rast.nmpdr.org/) (Brettin et al. 2015), GATU (https://4virology.net/virology-ca-tools/gatu/) (Tcherepanov et al. 2006), and Vgas (http://guolab.whu.edu.cn/vgas/) (Zhang et al. 2019). The predicted genes were based on RASTtk, and the results were validated using Vgas and GATU. In cases of conflict between RASTtk and the other two tools, the results were predicted by looking for results in the literature. Functional annotations of putative genes were evaluated using the online platforms Protein-Blast (https://blast.ncbi.nlm.nih.gov) (Bujak et al. 2020) and InterPro (https://www.ebi.ac.uk/interpro/) (Paysan-Lafosse et al. 2023). Gene function annotation was performed using BLAST-P (*e* = 0.05), while validation was performed using InterPro. Functional protein annotations were used instead of hypothetical proteins. The tRNAscan-SE (http://lowelab.ucsc.edu/tRNAscan-SE/) (Chan et al. 2021) was utilized for identifying the existence of transfer RNA (tRNA) genes. Antibiotic resistance genes were predicted using the Comprehensive Antibiotic Resistance Database (CARD). (Alcock et al. 2023; Finton et al. 2020). A circumferential graph of the phage was drawn by Proksee Server (https://proksee.ca/) (Grant and Stothard 2008).

### Genome comparison

A comparison of high similarity phage genomes was done using Easyfig 2.2.5 (https://mjsull.github.io/Easyfig/) (Sullivan et al. 2011). Similar phages to vB_AbaM_P1 and representative phage genome sequences of the three *Saclayvirus* families were compared using blast 2.12.0 + and visualized by Brig (threshold: *e* value of 1 × 10^–10^) (Alikhan et al. 2011). Based on the results of brig, the ORF correspondence of different genomes with vB_AbaM_P1 was compared. The amino acid sequences of the major capsid protein, portal protein, terminase large subunit, and DNA polymerase of vB_AbaM_P1 were compared with those of other *Saclayvirus* family phages in the protein database by BLASTp. The species with significant matches (with an *e* value lower than 0.05) to all four query proteins were selected, and all these sequences were downloaded as “FASTA (complete sequence)”. Subsequently, utilizing these sequences, phylogenetic trees were constructed using Molecular Evolutionary Genetic Analysis (MEGA) version 7.0 (Kumar et al. 2016).

### Neutrophil-deficient model

The neutrophil-deficient animal model was made by CTX with some modifications (Jeon et al. 2016). Briefly, Wistar rats were fixed, their tails were cleaned with 75% alcohol disinfectant, and then they were stimulated for tail vein exposure. To create the immunosuppressive animal models, the bacterium infection was preceded by injecting CTX through the tail vein at a dosage of 100 mg/kg 4 days in advance. Three days after the CTX injection, blood samples were collected from the tail vein to determine the count of whole blood cells.

### Protective effect of phage on rat models

To evaluate the protective effect of vB_AbaM_P1 on *A. baumannii* pneumonia in model rats, rats were divided into four groups: (I) control group (*n* = 10); (II) phage prevention and PBS treated (*n* = 10); (III) phage prevention and *A. baumannii* treated (*n* = 10); and (IV) PBS and *A. baumannii* treated (*n* = 10). Briefly, one day before the treatment of *A. baumannii*, model rats in groups II and III were pre-injected with 10^12^ PFU with vB_AbaM_P1 by intraperitoneal (i.p.) injection, and group IV was pre-injected with PBS. At the time of infection, rats were anesthetized by i.p. injection of pentobarbital sodium (50 mg/kg) in an experimental pneumonia model by airway drip injection of 50 μL of *A. baumannii* strain CGMCC 1.90331 at a concentration of 4 × 10^8^ CFU/mL (LD_100_). The survival rate of each group was measured up to seven days post-infection.

In a follow-up analysis based on the results of the phage protection rate in rats, the rats were once again categorized into four groups (*n* = 20) with the same treatment criteria. Rats were euthanized with pentobarbital sodium (150 mg/kg) at 12 h, 24 h, and 48 h after bacterial inoculation (*n* = 5 rats per group). Lung tissue and serum were collected under aseptic conditions.

### Bacterial counts

The collected left lung from each group (*n* = 5) were weighed and homogenized, diluted continuously with PBS, and counted viable bacteria on LB agar plates that contained ampicillin (50 μg/mL).

### Histological analysis

Rats were euthanized 48 h after infection, and one lobe of the right lung from each group (*n* = 5) was preserved in 4% (wt/vol) paraformaldehyde for 48 h. After that, it underwent paraffin embedding and sectioning, followed by hematoxylin–eosin (HE) staining. Optical microscopy was used to observe sections.

### Anti-oxidation capacity and cytokine determination

One lung lobe of the right lung was collected from each group (*n* = 5) at 12 h, 24 h, and 48 h for the determination of antioxidant capacity. The corresponding commercial kit purchased from Nanjing Jiancheng Bioengineering Institute (Nanjing, China) was used to detect superoxide dismutase (SOD, Jiangcheng, Nanjing, China, A001-3–2) activity and malondialdehyde (MDA, Jiangcheng, Nanjing, China, A003-1–2) counts in lung tissue.

For cytokine levels in serum, we used ELISA kits to detect the serum contents of tumor necrosis factor-α (TNF-α, Multisciences, Hangzhou, China, EK382), interleukin-1β (IL-1β, Multisciences, Hangzhou, China, EK301B), and interleukin-6 (IL-6, Multisciences, Hangzhou, China, EK306).

## Statistical analysis

GraphPad Prism software was utilized to perform statistical analyses on the data. The survival rate was calculated by Log-rank tests, and the others were expressed as means ± SD. Statistical analysis was conducted using a one-way ANOVA, followed by Tukey’s multiple comparison test. *P*-values less than 0.05 (*), 0.01 (**), 0.001 (***), and 0.0001(****) were considered significant.

### Accession number

The complete nucleotide sequence of the vB_AbaM_P1 genome has been deposited in NCBI GenBank (accession no: OL960030).

## Results

### Phage host range

The strains utilized in this experiment for phage host profiling experiments are listed in Table 1. Among the 35 tested strains, vB_AbaM_P1 exhibited a wide range of hosts and successfully lysed five strains of *A. baumannii *(EOP > 0.1).

**Table 1:** Host range determination of vB_AbaM_P1

Species	Strain	Reference	Host range^a^	EOP^b^
*Acinetobacter baumannii*	AB-R1	OQ881017	-	-
*Acinetobacter baumannii*	AB-S3	OQ881018	-	-
*Acinetobacter baumannii*	AB-R4	OQ881020	+ +	0.9
*Acinetobacter baumannii*	ABR5	CGMCC 1.90331	+ +	1
*Acinetobacter baumannii*	AB-R6	OQ881019	-	-
*Acinetobacter baumannii*	AB-R7	OR186691	-	-
*Acinetobacter baumannii*	AB-R8	OR186693	+	< 0.1
*Acinetobacter baumannii*	AB-R9	OR186692	+ +	0.6
*Acinetobacter baumannii*	AB-R11	OR186694	-	-
*Acinetobacter baumannii*	AB-R12	OR186698	+	< 0.1
*Acinetobacter baumannii*	AB-R13	OR186696	+	< 0.1
*Acinetobacter baumannii*	AB-S14	OQ881027	-	-
*Acinetobacter baumannii*	AB-R15	OR186697	-	-
*Acinetobacter baumannii*	AB-R16	OQ881023	-	-
*Acinetobacter baumannii*	AB-R17	OR186699	-	-
*Acinetobacter baumannii*	AB-R18	OR186700	+ +	0.5
*Acinetobacter baumannii*	AB-R19	OR186710	-	-
*Acinetobacter baumannii*	AB-R20	OR186714	+ +	0.7
*Acinetobacter baumannii*	AB-R21	OR186708	-	-
*Acinetobacter baumannii*	AB-S22	OQ881025	-	-
*Acinetobacter baumannii*	AB-R24	OQ881024	+	< 0.1
*Acinetobacter baumannii*	AB-R25	OQ881025	-	-
*Acinetobacter baumannii*	AB-R26	OR186719	-	-
*Acinetobacter calcoaceticus*	AC-01	OQ880922	-	-
*Acinetobacter calcoaceticus*	AC-02	OQ880921	-	-
*Klebsiella pneumoniae*	KP-S1	OR020689	-	-
*Klebsiella pneumoniae*	KP-S2	OR020694	-	-
*vibrio harveyi*	VH-4	OQ880823	-	-
*vibrio harveyi*	VH-6	OQ880824	-	-
*vibrio harveyi*	VH-7	OQ880822	-	-
*vibrio harveyi*	VH-8	OQ880825	-	-
*Salmonella typhimurium*	ST-1	CMCC50115	-	-
*Salmonella typhimurium*	ST-2	CMCC50220	-	-
*Salmonella hirschfeldii*	SH-1	CICC21512	-	-
*Salmonella paratyphi A*	SP-1	CICC21501	-	-

^a^Phage infectivity: +  + , clear; + , translucent; − , no plaque

^b^EOP was calculated as the titer of the phage (PFU/ml) in the test strain divided by the titer of phage (PFU/ml) in the host strain CGMCC 1.90331

### Morphology of phage vB_AbaM_P1

**Fig. 1:**
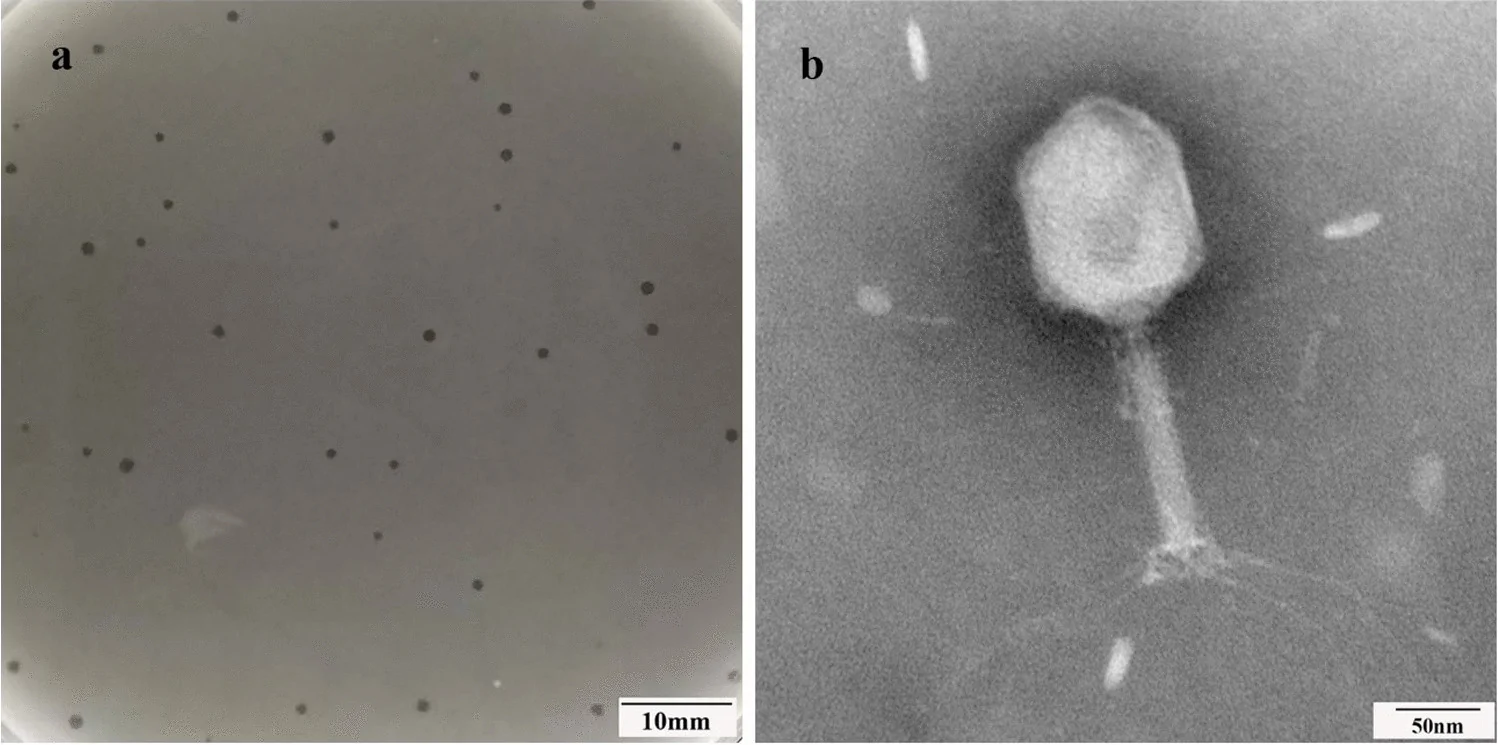
Patchy and viral morphology of vB_AbaM_P1. **a** Plaque morphology of vB_AbaM_P1 cultured on double agar plates for 12 h. **b** Transmission electron micrograph of vB_AbaM_P1 negatively stained with 2% w/v uranyl acetate

On the double agar plate, each phage could form a round and clear plaque of about 1 mm in diameter (Fig. 1a), with *A. baumannii strain* CGMCC 1.90331 as the host bacterium. According to the analysis using transmission electron microscopy, it was observed that the phage vB_AbaM_P1 had a head with a diameter of approximately 70 nm that was evenly spaced. Additionally, the phage had a tail that measured 140 nm in length and 20 nm in width (Fig. 1b). vB_AbaM_P1 had a morphological appearance that resembled the *Saclayvirus* phage previously described (Essoh et al. 2019).

## Phage adsorption rate, burst size, and latent period

**Fig. 2:**
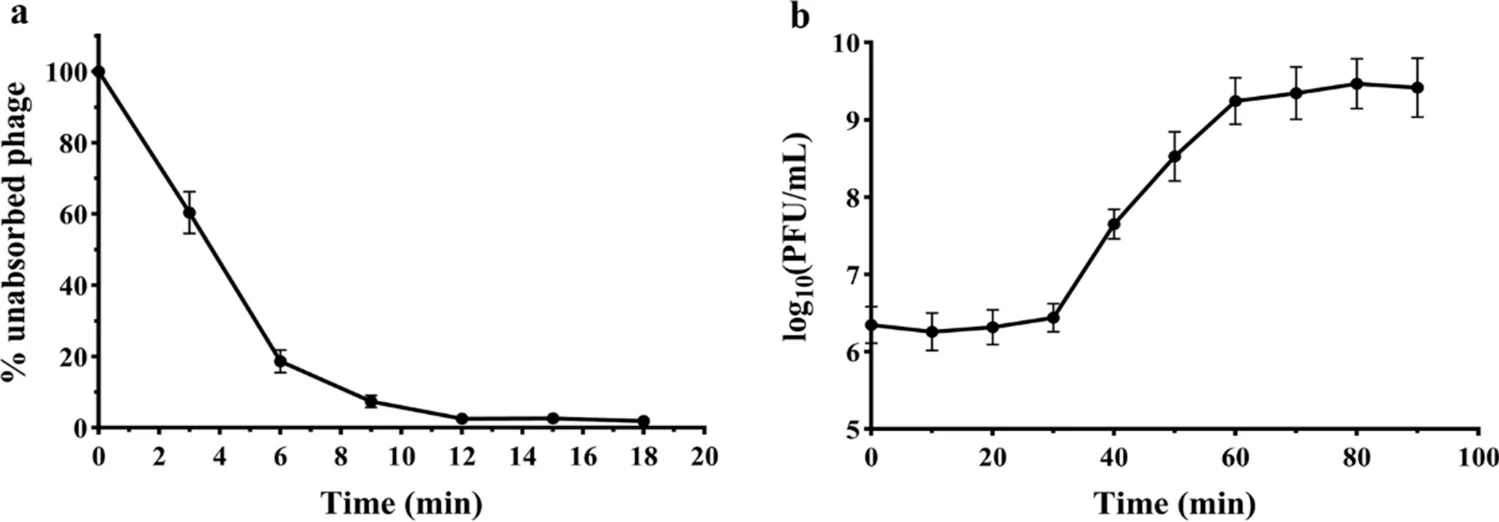
Biological characterization of phage vB_AbaM_P1. **a** The adsorption rate of vB_AbaM_P1 to host bacteria CGMCC 1.90331 was assayed at 3 min intervals for 18 min continuously at 37 °C with an MOI of 0.1. **b** One-step growth curve of vB_AbaM_P1 with AB-5 as the host bacterium, error bars represent standard deviation of three independent experiments

More than 95% of P1 adsorbed and infected the host bacteria in about 12 min (Fig. 2a). The one-step growth curve of the phage reflected two important parameters: incubation period, and burst size (Fig. 2b). vB_AbaM_P1 had an incubation and growth period of 30 min each, accompanied by a burst size of 788 PFU per cell.

### Antimicrobial activity of phage

**Fig. 3:**
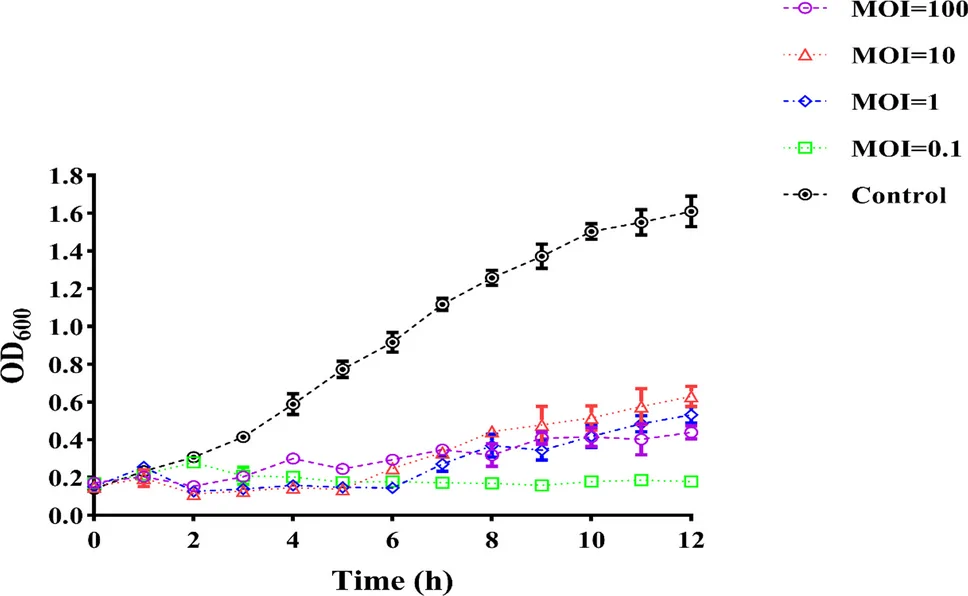
Activity assay of vB_AbaM_P1 against *A. baumannii* in vitro. *A. baumannii* was infected at MOI of 100, 10, 1, 0.1 or the same volume PBS for up to 12 h. This trial was replicated three times, and the results were presented as the average ± standard deviation

In the analysis of phage lysis stability, vB_AbaM_P1 can inhibit bacterial growth at all MOIs (MOI = 100, 10, 1, 0.1), which is significantly different from the bacterial culture with PBS. When MOI = 0.1, the phage has the best antibacterial time and effect (Fig. 3).

### Characterization of the phage vB_AbaM_P1 genome

**Fig. 4:**
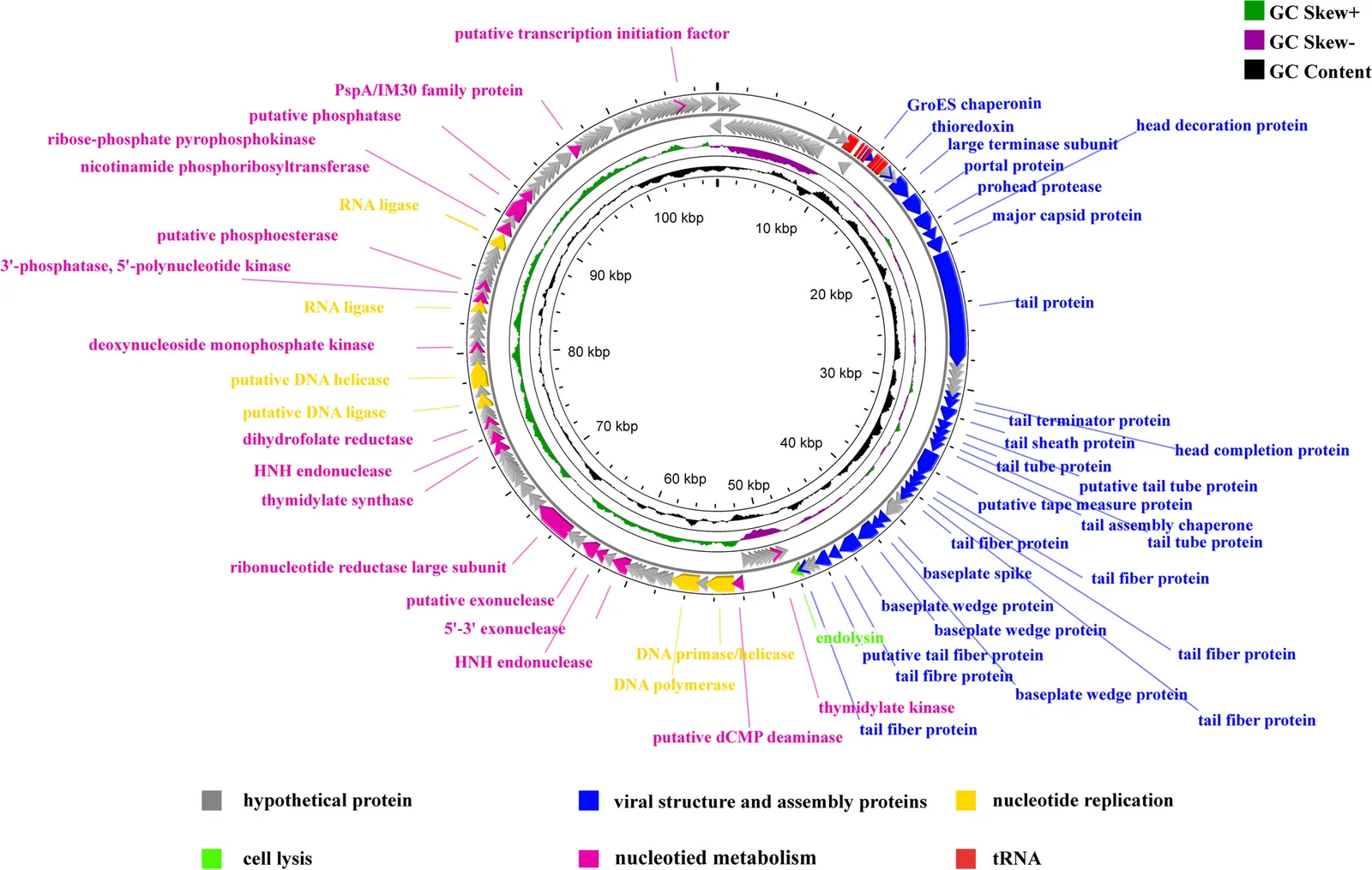
Genome analysis of vB_AbaM_P1. The external loop displays the open reading frame (ORF) of the phage and the direction in which it is transcribed. Inner ring indicates GC content (black) and GC skew (green and purple)

The phage genome is extracted, and the Illumina sequencing platform is utilized for genomic sequencing. The whole genome sequence of vB_AbaM_P1 contains 107,537 bp with the COS packaging mechanism, 1153 bp of terminal repeat sequence, and 37.68% G + C content. A total of 191 gene regions were predicted (177 ORFs plus 14 tRNAs); 31 of them were leftward-directed and 160 were rightward-directed (Table S1). Among them, 51 of the predicted ORFs have designated functions. The putative function can be divided into five components: viral structure and assembly proteins (27), nucleotide replication (6), cell lysis (1), nucleotide metabolism (17), and tRNA (14). The remaining 126 ORFs were predicted to be hypothetical proteins (Fig. 4), and their functions need to be further investigated.

In the phage genome, 14 tRNA genes were inferred, of which 10 tRNAs had identical predictions in RASTtk and tRNAscan-SE, and the other four tRNAs (Met-CAT, Asn-GTT, Cys-GCA, and Leu-TAG) were predicted by tRNAsan-SE. In addition, the results of the screen using the CARD database showed that the phage vB_AbaM_P1 genome did not contain genes encoding antibiotic resistance-associated products. The complete vB_AbaM_P1 sequence was uploaded to GenBank with accession number OL960030 (https://www.ncbi.nlm.nih.gov).

### Analysis of comparative genomes

**Fig. 5:**
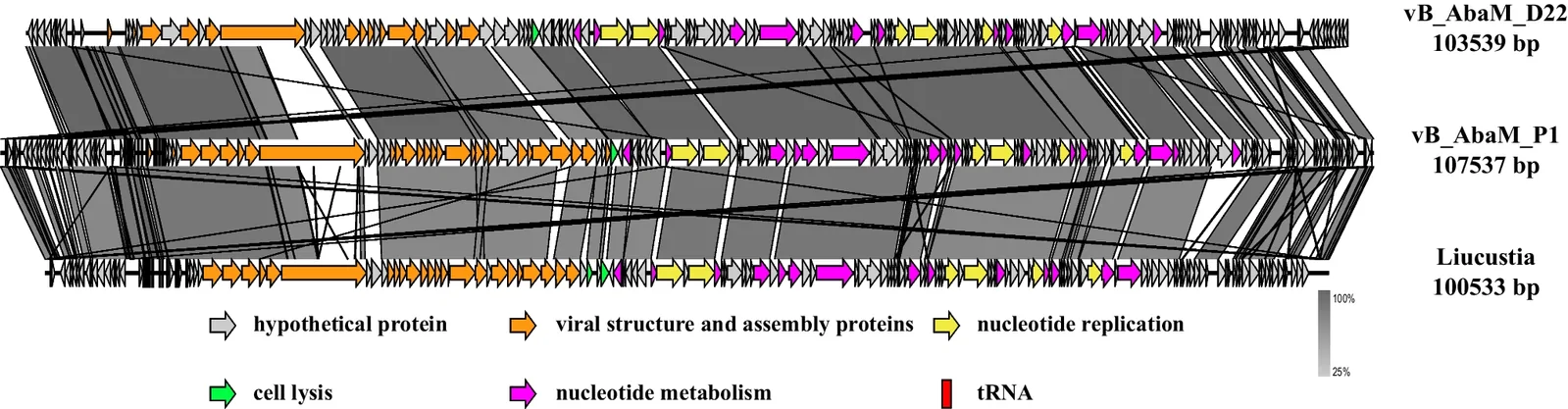
Genome alignment of vB_AbaM_P1 with vB_AbaM_D22 and Liucustia. Different arrows indicate ORFs and transcription direction. As shown in the legend, different colors represent genes with various functions, based on their function. Similarities between genomes are indicated by gray lines

Nucleotide BLAST was utilized to perform similarity matching of the entire genome of vB_AbaM_P1. The results showed the highest sequence similarity and length coverage with phage vB_AbaM_D22 (~ 95.32% percent identity and ~ 78% query coverage). It also had a high similarity with phage Liucustia (~ 88.78% identity and ~ 62% query coverage). Of the 177 ORFs predicted in vB_AbaM_P1, 142 were similar to vB_AbaM_D22 (Supplementary Table S2). Similarly, vB_AbaM_P1 had 135 ORFs similar to Liucustia (Supplementary Table S3). Of the 51 ORFs that have been putatively functional, 33 ORFs are shared with vB_AbaM_D22, and 37 ORFs are shared with Liucustia (Fig. 5).

**Fig. 6:**
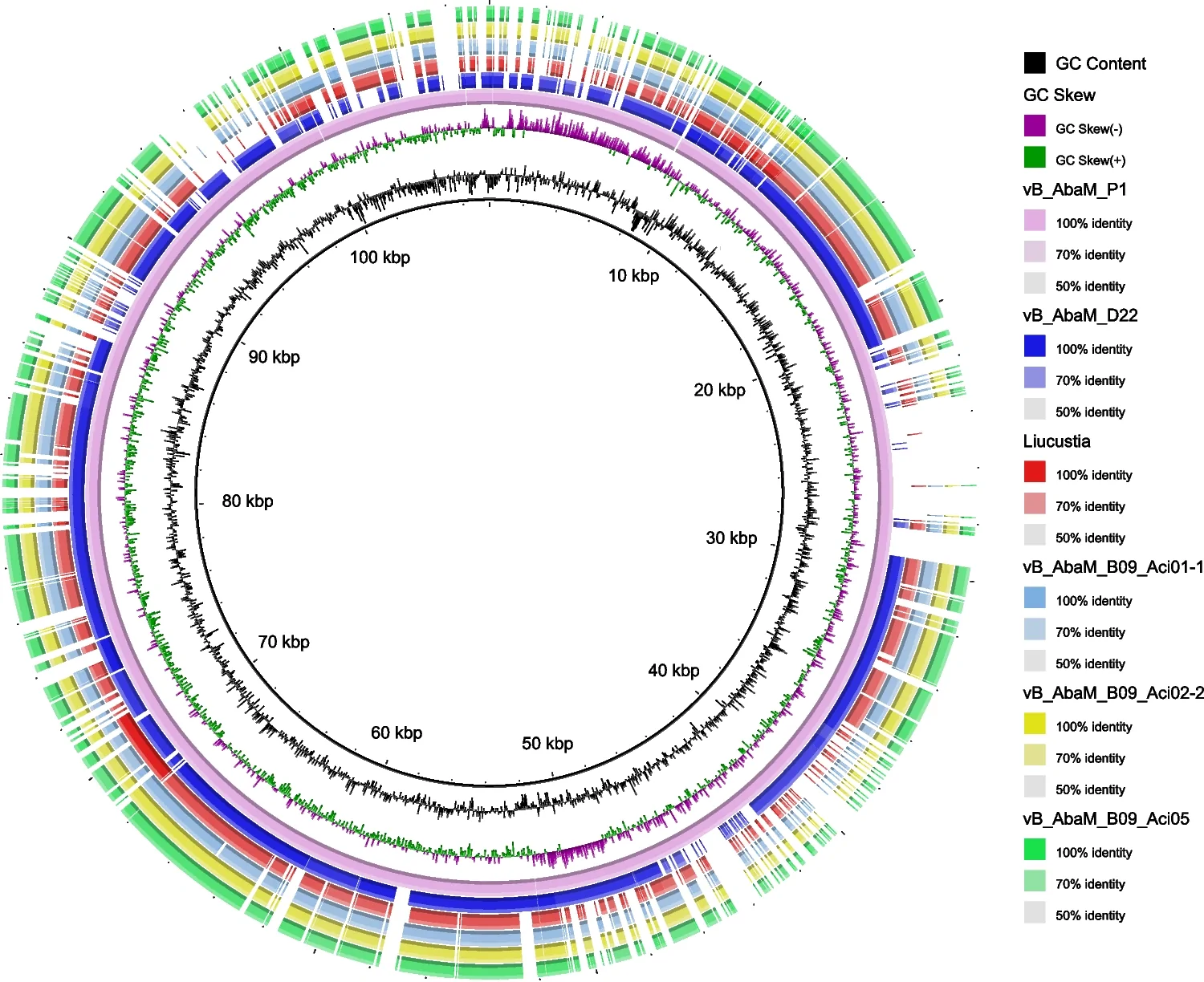
Comparison of vB_AbaM_P1 with other homologous phage genomes. Depending on the brightness of the color, it represents 50 to 100% similarity. The rings in the inner circle represent GC content and GC skew, respectively

vB_AbaM_P1 was further compared with the *Saclayvirus* phage representative strain (vB_AbaM_B09_Aci02-2, vB_AbaM_B09_Aci01-1, and vB_AbaM_B09_Aci05) in the ICTV database, and the genome of vB_AbaM_P1 showed high sequence homology and coverage with the genome of representative strains of the *Saclayvirus* family (Fig. 6). Phylogenetic analysis of the conserved protein sequences of vB_AbaM_P1 (DNA polymerase, Supplementary Fig. S1; major capsid protein, Supplementary Fig. S2; portal protein, Supplementary Fig. S3; large terminase subunit, Supplementary Fig. S4) revealed that vB_AbaM_P1 is closely related to vB_AbaM_D22. Although vB_AbaM_D22 has been classified as a *Saclayvirus* phage in the NCBI database, literature describing its biological and morphological properties is lacking.

### Phage protection against *A. baumannii* pneumonia in neutrophil-deficient model

To evaluate the effect of CTX on immune cells in rats, blood samples were collected for whole blood cell counts after CTX injection. The white blood cells and immune cell components in the blood were much lower than normal values (Table S4).

**Fig. 7:**
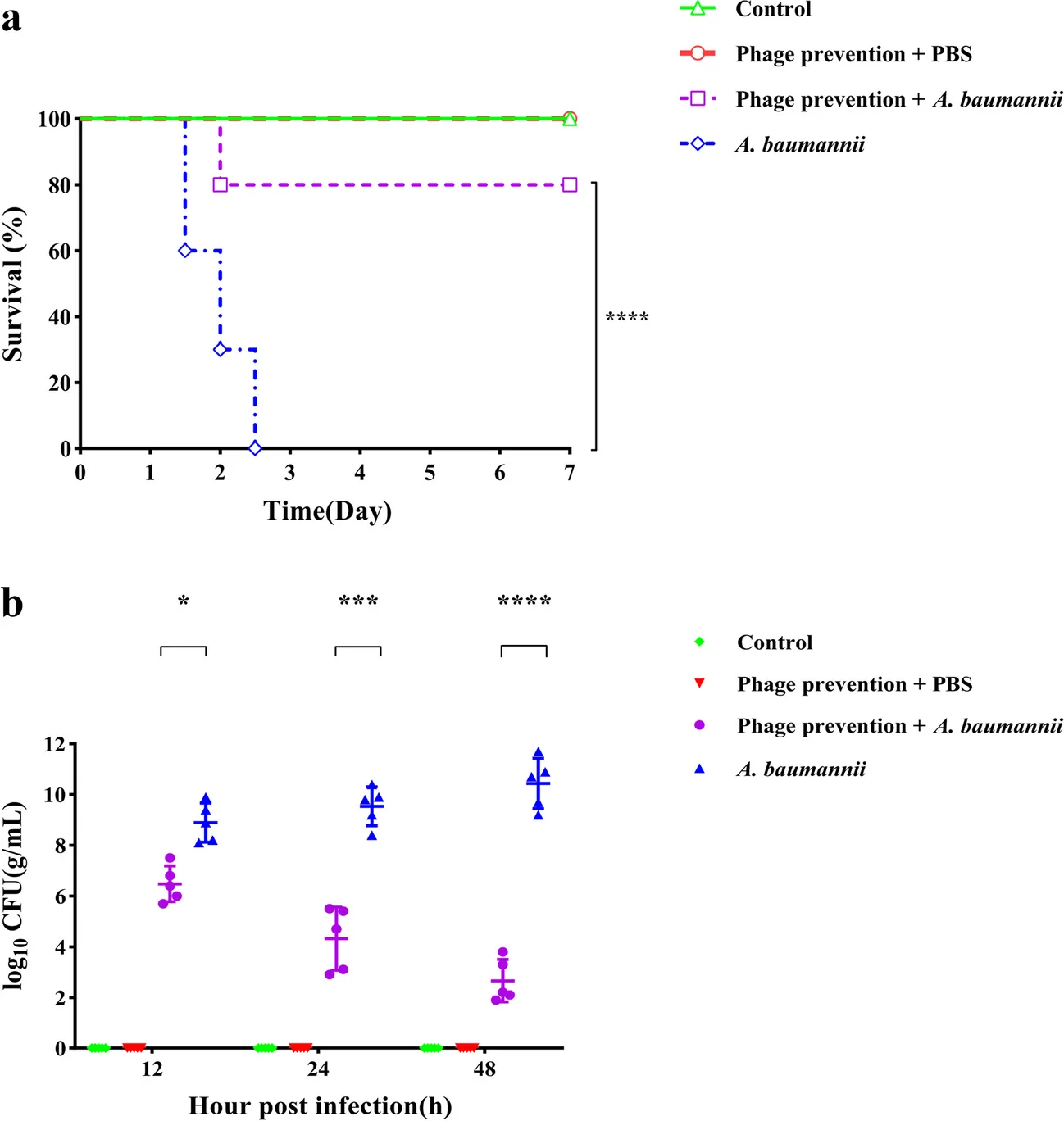
Effect of vB_AbaM_P1 on prevention of *A. baumannii* pneumonia in a neutrophil-deficient model. **a** 7-day survival of rats. Male Wistar rats were divided into four groups (*n* = 10): control group, phage and PBS treatment, phage prevention and *A. baumannii* treatment, *A. baumannii* treatment. **b** Bacterial counts in lung tissue at 12 h, 24 h, and 48 h post-infection in different treatment groups. The bacterial counts of each group were compared using a one-way ANOVA. (*P < 0.05, ***P < 0.001, ****P < 0.0001)

The protective effect of vB_AbaM_P1 on neutrophil-deficient models with *A. baumannii* pneumonia was evaluated by measuring the survival rate of rats after prophylactic application of vB_AbaM_P1. As shown in Fig. 7a, all rats infected with *A. baumannii* died within 60 h. In rats protected by prophylactic application of vB_AbaM_P1, the survival rate at day 7 was 80%, and all the rats in the phage and PBS treatment groups, including the control group, survived.

### Bacterial counts

The number of bacteria present in lung tissue samples of each group measured at 12 h, 24 h, and 48 h post-infection is shown in Fig. 7b. Compared with the bacterial infection group, the growth of *A. baumannii* in the prophylactic application group was inhibited at 12 h, and the number of *A. baumannii* decreased significantly after 24 h, with a further decrease after 48 h. With the prolongation of the infection, the colonization of bacteria in the bacterial infection group had gone out of control. *A. baumannii* were not found in the phage and PBS treatment groups, or in the control group.

### Histology

**Fig. 8:**
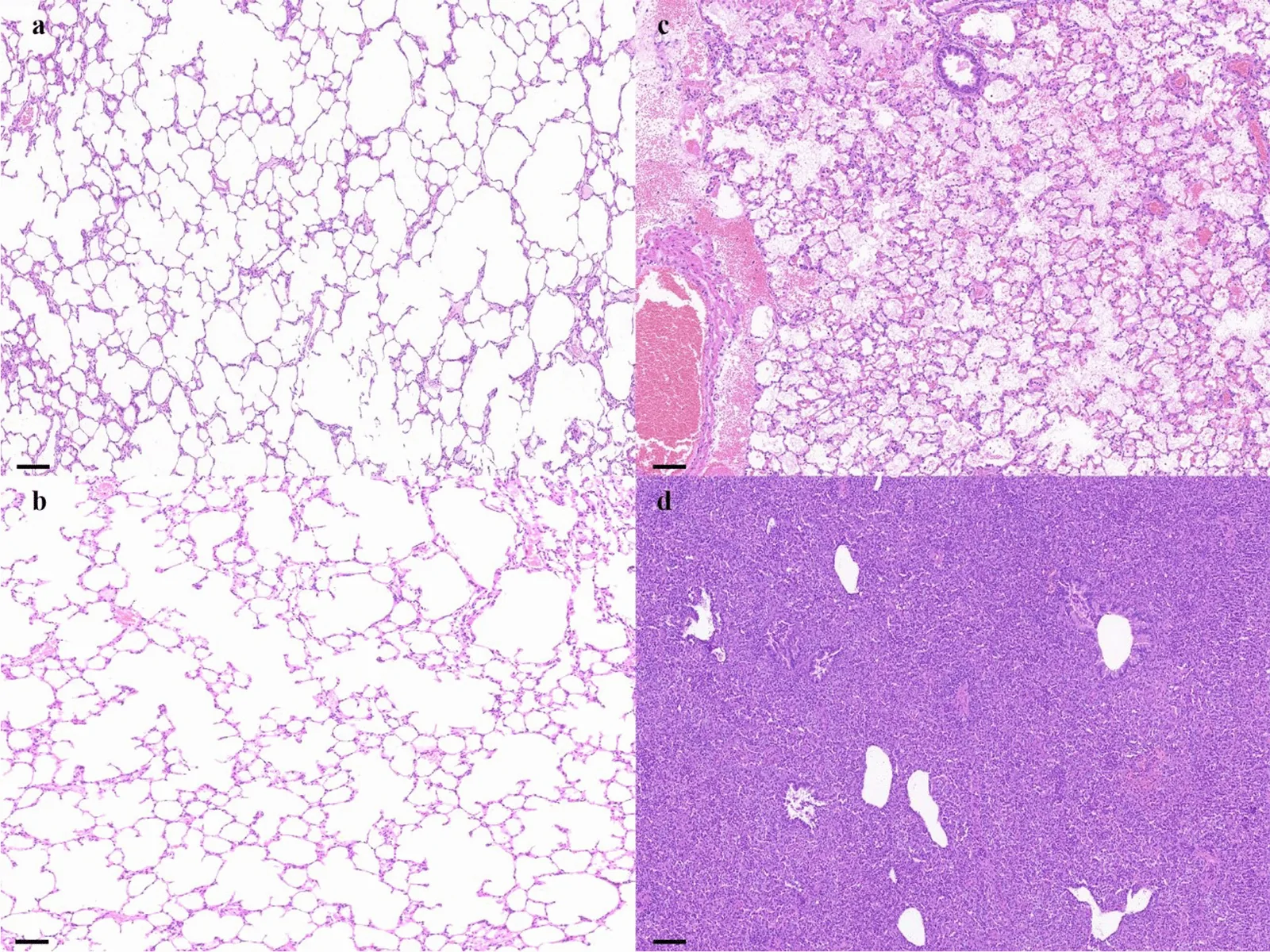
Histological analysis of lung sections across different treatment groups. Rats were euthanized 48 h after infection. **a** Control group; **b** phage and PBS treatment; **c** phage prevention and *A. baumannii* treatment; and **d** *A. baumannii* treatment. The stained sections were visualized at 100 × magnification. The scale bars represent 100 μm

In order to evaluate the protective effect of phage prophylactic application on lung tissue in a neutrophil-deficient model, HE staining was used to observe the alterations in lung tissue samples (Fig. 8). At 48 h, the alveolar structure disappeared in the bacterial infection group. Compared with bacterial infections, phage prevention can effectively reduce lung tissue damage caused by *A. baumannii*. The Phage and PBS treatment groups had similar results to the control group.

### SOD and MDA activity

**Fig. 9:**
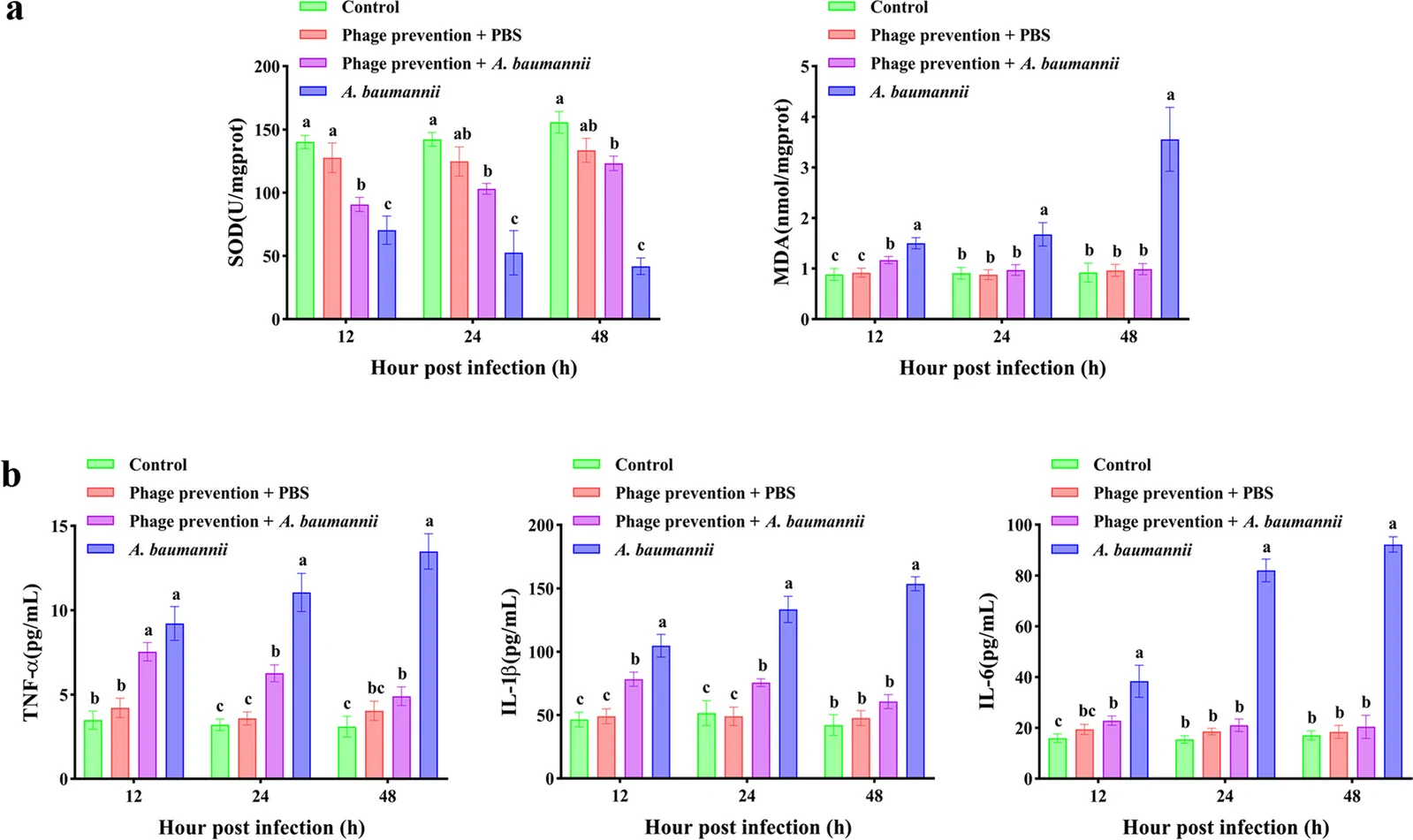
Lung tissue oxidative stress and serum cytokine levels. **a** SOD activity and MDA levels. **b** Changes in cytokines TNF-α, IL-1β, and IL-6 in serum. Data is expressed as mean ± SD (*n* = 5). Columns with distinct letters are significantly different (*P* < 0.05)

Oxidative stress indicators found in lung tissue were shown in Fig. 9a. The SOD level in the bacterial infection group decreased continuously, while that in the prophylactic application group remained stable. Compared with the *A. baumannii* infection group, the prophylactic application reduced MDA contents in the lung tissue. No differences were found in the SOD or MDA between the prophylactic application group and the control group.

### Cytokine analysis

Pulmonary inflammation was closely related to the level of cytokines (Fig. 9b), With the extension of time, the level of cytokines in the prophylactic application group decreased gradually, while the cytokines in the bacterial infection group reached their highest value at 48 h. There were no notable disparities in cytokine levels between the phage and PBS treatment groups and the control group.

## Discussion

*Acinetobacter baumannii*, a Gram-negative pathogen that opportunistically infects individuals, has developed numerous mechanisms to resist drugs, particularly exhibiting a rapid rise in resistance to carbapenem antibiotics (Huang et al. 2019), which increases its pathogenic potential and poses a great threat to patients and healthcare workers. *A. baumannii* is recognized as a globally important hospital pathogen (Niu et al. 2018; Vazquez-Lopez et al. 2020). Regrettably, nearly all hospital-acquired infections, whether acute or chronic, that are caused by *A. baumannii* are linked to antibiotic resistance. As antibiotic resistance becomes more prevalent, more and more researchers are refocusing their efforts toward phage therapies that target superbugs, and a large number of studies have proven that the use of phages as an alternative to antibiotics is promising (Gong et al. 2021; Kortright et al. 2019). However, unlike previous studies that focused on phage therapies for post-infection bacteria, this study mainly focused on the prophylactic application of phages to prevent bacterial infections.

We isolated a novel lytic phage vB_AbaM_P1, from the hospital effluent and purified the phage to obtain a uniformly sized phage spot. According to the NCBI classification, vB_AbaM_P1 belongs to the *Saclayvirus* family. As observed by transmission electron microscopy, the morphology and size of the phage showed that vB_AbaM_P1 has a long crystalline head and a constricted tail with spikes and caudal fibers. Sequence alignment of the genome provides further support that vB_AbaM_P1 belongs to this family (Essoh et al. 2019). In common with other phages of the same family, vB_AbaM_P1 has a broad host spectrum (Asif et al. 2020; Merabishvili et al. 2014). The vB_AbaM_P1 showed an optimal MOI of 0.1 against the *A. baumannii* strain CGMCC 1.90331, and the phage did not induce resistance in the host bacteria throughout the 12-h experiment.

The growth characteristics of phages, mainly including adsorption rate, latency, and burst size, are key factors in determining their application potential. vB_AbaM_P1 rapidly adsorbed to the host in approximately 12 min, indicating rapid phage binding to the host receptor. The vB_AbaM_P1 had a moderate latency period of 30 min and a high burst size of 788 particles per cell. Compared to other phages in the *Saclayvirus* family, vB_AbaM_P1 exhibits a longer latency period and a higher burst size: TAC1 has a latency period of 15 min and a burst size of 454 PFU/cell (Asif et al. 2020); vB_AbaM_Acibel004 has a latency period of 27 min and a burst size 125 PFU/cell (Merabishvili et al. 2014). Latency period and burst size are positively correlated, with a longer latency period accompanied by a larger burst size (Shao and Wang 2008).

The vB_AbaM_P1 contains linear double-stranded DNA with a genome size of 107,537 base pairs and direct terminal repeats measuring 1153 base pairs. The genomic features are consistent with phages in the *Saclayvirus* family, all genome lengths are larger than 10 kb, including direct terminal repeats (Essoh et al. 2019). The vB_AbaM_P1 genome encodes up to 14 tRNAs located between 9702 and 12,903. tRNAs are commonly distributed in the dsDNA phage genome, with lytic phages encoding more tRNAs than temperate phages (Bailly-Bechet et al. 2007). It is believed that the tRNA present in the phage genetic material helps to offset the variation in codon utilization between the phage and the host, ultimately decreasing the time required for incubation and enhancing the rate of reproduction, thus increasing the efficiency of phage infection (Santos et al. 2011). The vB_AbaM_P1 genome does not contain the genes that encode enzymes involved in lysogeny (e.g., integrase), inferring that vB_AbaM_P1 is a lytic phage. Screening against the CARD database did not match the gene products encoding antibiotic resistance-associated genes in the vB_AbaM_P1 genome.

In the infection cycle of a double-stranded DNA phage, the lysis process mainly implicates two proteins, endolysin and holin (Young 2013). vB_AbaM_P1 is a lytic bacteriophage that should produce lytic proteins and cause disruption of the bacterial cell wall. ORF66 encodes an endolysin (IPR: 023346), belongs to a family of the lysozyme-like domain superfamily found in glycosyl hydrolases and transglycosylases, which is highly similar to vB_AbaM_D22 (~ 98.40%) and less similar to Liucustia (~ 56.80%). The gene encoding holin is not predicted in the genome of vB_AbaM_P1, and in tailed phages, holin is often located near endolysin (Asif et al. 2020). The ORF54 of Liucustia was presumed to be holin, similar to ORF63 of vB_AbaM_P1 (~ 86.47%). ORF63 was predicted to be a hypothetical protein containing two transmembrane structural domains. Since holin has a transmembrane structural domain, it can be inferred that ORF63 may encode holin. In addition, bacterial lysis can be achieved through inhibition of peptidoglycan synthesis and inhibition of host-specific enzymes (Asif et al. 2020), and the mechanism of host cleavage of vB_AbaM_P1 needs to be further investigated.

In vB_AbaM_P1, the positive strand contains the genes that encode the main components of the double-stranded DNA-tailed phage, including terminase, portal protein, major capsid protein, and tail fiber protein. ORF37 was predicted to be the major capsid protein (IPR005564) and was highly similar to ORF20 of vB_AbaM_D22 and ORF29 of Liucustia (~ 99.45% and ~ 84.66%). Major capsid proteins assemble to form an icosahedral capsid with a T = 7 symmetry (Dokland and Murialdo 1993; Katsura and Kobayashi 1990). It contributes to the stabilization of the compact structure of the DNA molecule in phage heads (Katsura 1989). In addition, ORF35 presumably encodes a prehead protease, which is important for phage capsid morphogenesis, and the maturation of capsid protein precursors ensures the stability of nucleic acid-free precursors. The gene for the prehead protease is often located around the major capsid protein gene, and, in some cases, may be fused to the major capsid protein gene. The two genes in vB_AbaM_P1 are in close proximity to each other (ORF35 and ORF37), separated by a head-modifying protein (Liu and Mushegian 2004). During phage packaging, portal proteins are involved in the DNA packaging and delivery process by forming a twelve-position symmetric ring (Daniel et al. 2007; Prevelige and Cortines 2018). During packaging and injection, DNA enters and leaves through this unique ring. After the DNA is packaged, the tail completes the assembly of the viral particle by attaching to this ring (Casjens 2011). ORF34 of vB_AbaM_P1 was predicted to be a portal protein and was highly similar to ORF26 of Liucustia (~ 87.10%). ORF33 may encode a terminase large subunit (HHpred: 5OE8_B), which contains a C-terminal domain (IPR035421) that is highly similar to ORF16 of vB_AbaM_D22 and ORF25 of Liucustia (~ 99.61% and ~ 91.26%). The packaging of the phage genome is highly conserved, and after replication of the genes, the genome forms a tandem in a head-to-tail fashion, is digested into a unit-length genome by terminase, and is promoted by ATP hydrolase to translocate into the procapsid (Yang et al. 2017). The tail fiber proteins of the tailed phage make the phage identify and adhere to the host’s surface and inject nucleic acids (Maffei et al. 2021). Several tail fiber protein genes were predicted in the genome of vB_AbaM_P1 (ORF51, 52, 53, 54, 61, 62, 65). The tail fiber protein of bacteriophages is believed to have a significant impact on the recognition of hosts and the determination of the range of hosts (Yang et al. 2018). Furthermore, baseplate proteins and tail spike proteins functioned in both phage adsorption and receptor binding (Evseev et al. 2020; Li et al. 2016).

Several proteins associated with nucleic acid metabolism were predicted in the genome of vB_AbaM_P1. Among them are multiple phosphotransferases (ORF68, 119, 126, 128, 137, 139, 140), which facilitate the transfer of phosphate groups from donor molecules with high energy to substrate molecules, thereby enhancing the activity of the substrate molecules ORF109 was predicted to be a dihydrofolate reductase (IPR012259, HHpred: 4M7U_A), highly similar to ORF86 of vB_AbaM_D22 and ORF95 of Liucustia (~ 86.70% and ~ 90.96%). Dihydrofolate reductase is a key enzyme in folate metabolism, catalyzing the NADPH-dependent reduction of dihydrofolate to tetrahydrofolate, which is a crucial process in the production of glycine, purine, and phosphate deoxythymine (DNA precursors) and also important in the conversion of deoxyuridine monophosphate to deoxythymine monophosphate (Trimble et al. 1988). ORF75 presumably encodes a deoxycytidylate deaminase (dCMP deaminase, IPR015517, HHpred: COG2131), which is highly similar to ORF56 of vB_AbaM_D22 (~ 100%). dCMP deaminase hydrolyzes deoxycytidylate mono phosphate to deoxyuridine monophosphate (dUMP), which provides nucleotide substrates for thymidylate synthase and plays an important role in DNA synthesis (Hou et al. 2008). ORF105 encodes a thymidylate synthase (IPR045097, HHpred: 6KP7_B), similar to ORF83 of vB_AbaM_D22 and ORF92 of Liucustia (~ 90.85% and ~ 80.37%). The enzyme plays a crucial role in maintaining a balanced supply of the four DNA precursors during normal DNA replication. Abnormalities in the enzyme’s activity can disrupt the regulatory process and result in different biological and genetic abnormalities, including thymineless death (Kaneda et al. 1990).

Concerning nucleic acid replication, ORF76 may encode a DNA helicase (IPR027032, HHpred: 6N7I_E), similar to ORF57 of vB_AbaM_D22 and ORF65 of Liucustia (~ 99.68% and ~ 93.50%). ORF76 functions as a T7-like helicase in mitochondrial DNA, facilitating the ATP-dependent untangling of double-stranded DNA in the 5′ to 3′ orientation. To initiate the untangling process, it necessitates single-stranded 5′ DNA and a brief 3′ tail (Korhonen et al. 2003). ORF115 is predicted to be another DNA helicase (IPR000212), similar to vB_AbaM_D22’s ORF92 and Liucustia’s ORF101 (~ 99.36% and ~ 86.86%), facilitating the ATP-driven separation of double-stranded DNA into single-stranded DNA. ORF78 is predicted to encode a DNA polymerase (IPR002298, HHpred: COG0749) that is similar to ORF59 and ORF67 of Liucustia (~ 97.38% and ~ 91.70%), respectively. Two potential domains were detected within this gene: the DNA-directed DNA polymerase, family A, palm domain (IPR001098), and the 3′ to 5′ exonuclease domain (IPR002562), which facilitate accurate DNA replication and hydrolyze unpaired or mismatched nucleotides, respectively. ORF113 potentially encodes a DNA ligase (HHpred: 7OBN_A) which catalyzes the creation of an internucleotide ester bond between phosphate and deoxyribose, thereby joining two DNA fragments. It is active during DNA replication, repair, and recombination processes. ORF113 contains two domains: DNA ligase, ATP-dependent, central (IPR012310), and DNA ligase, OB-like domain (IPR029319). It is similar to ORF90 of vB_AbaM_D22 and ORF99 of Liucustia (~ 98.87 and ~ 89.55%). ORF125 potentially encodes a T4 RNA ligase (HHpred: 5TT6_A), similar to ORF102 of vB_AbaM_D22 and ORF110 of Liucustia (~ 99.67% and ~ 85.15%). ORF136 was also predicted to be an RNA ligase (HHpred: 2HVQ_A), similar to ORF115 of vB_AbaM_D22 and ORF121 of Liucustia (~ 61.83% and ~ 62.60%). RNA ligases mend RNA strand breaks within DNA: RNA and RNA:RNA but do not work in DNA: DNA duplexes (Nandakumar et al. 2006). HNH endonucleases are common in phage genomes (Kot et al. 2014) and are sometimes expressed to remove competition from other phages (GoodrichBlair and Shub 1996). Two HNH endonuclease-encoding genes (ORF88 and 106) were predicted in the vB_AbaM_P1 genome, and in the Liucustia genome, only ORF77 was predicted to have a similar protein function to ORF88 (~ 87. 80%).

Interestingly, we found that many ORFs of vB_AbaM_P1 showed similarity to vB_AbaM_D22 and Liucustia (Fig. 5), which had low homology (< 25%) at 20 to 30 kb. vB_AbaM_P1 has high coverage with other phage genomes (Fig. 6), again with a concentrated low threshold region between 20 and 30 kb, which corresponds to a gene that may encode a tail structural protein that may influence phage movement patterns.

We used CTX to produce neutrophil-deficient models with a more efficient tail vein injection and successfully obtained neutrophil-deficient rats. There was a significant decrease in the quantity of immune cells in the model animals’ blood. The immune system has a crucial function in eliminating phages in animals and humans. In the initial stage of phage entry into the body, phage clearance is mainly carried out by the innate immune system, which is mainly completed by phagocytes (Geier et al. 1973; Hodyra-Stefaniak et al. 2015). In immunodeficiency, phages can exist in the body for a longer time, which makes phages more effective in preventing infection. Phage prophylaxis has better potential in immunocompromised conditions.

Prophylactic application protected model rats from *A. baumannii* pneumonia. The findings indicated that prophylactic application could successfully decrease the death of rats caused by *A. baumannii* pneumonia. The prophylactic application significantly decreased the quantity of *A. baumannii* bacteria in lung tissue, as determined by bacterial counting. This showed that prophylactic application of phages could stop *A. baumannii* from getting into model animals. These results are comparable to those observed in previous research (Yen et al. 2017). The lungs are the site of gas exchange and severe lung damage caused by bacterial infection is often lethal. Prophylactic application of phages reduced the inflammatory response of the lung tissue caused by *A. baumannii* significantly and reduced lung tissue damage in rats.

Under normal circumstances, the body typically maintains a relatively stable level of reactive oxygen species (ROS), which are intermediates in regular biochemical processes (Ye et al. 2021). In pathological circumstances, due to the production and removal of ROS, a normal balance is lost, which often causes ROS damage to the body. SOD is an enzyme that scavenges ROS and reflects the ability of the body to resist ROS, which greatly contributes to upholding the equilibrium of oxygen metabolism within the body. MDA is the end product of ROS-damaged cells, which can reflect the severity of the injury (Li et al. 2020a). Our results showed that prophylactic application reduced the decrease in SOD caused by *A. baumannii* infections, suggesting that ROS production decreased and the infection was alleviated. At the same time, when compared to the infection group, the MDA content in the prophylactic application group changed only slightly, suggesting a lesser degree of tissue damage. These findings support the idea that the application of phage prophylactics can reduce ROS production as well as the degree of tissue damage. It is worth noting that in the early stages of infection, SOD and MDA changes were not obvious, but after the formation of a serious infection, both of them changed significantly, which illustrates that *A. baumannii* in the body can, and likely will, cause rapid and serious damage throughout the body.

Cytokines play a crucial role in regulating the immune response by maintaining immune system balance and facilitating communication between various immune cells (Li et al. 2020b). Macrophages have a significant role in the fight against infection and can release various cytokines. We measured TNF-α, IL-1β, and IL-6 to confirm the immune system’s activation and the level of infection within the body. The findings indicated that, in comparison to the *A. baumannii* infection group, the prophylactic application could reduce the concentration of three cytokines caused by infection, suggesting that the immune system response caused by the original infection was reduced.

## Supplementary Information

Below is the link to the electronic supplementary material.

Supplementary file1 (PDF 507 KB)

Supplementary file2 (XLSX 49 KB)

## Data Availability

All relevant data are included in this article and supplementary materials. The nucleotide sequence of the phage genome has been deposited in NCBI GenBank, which can be queried by the accession number OL960030.
