# Supplementary material for: A novel Saclayvirus Acinetobacter baumannii phage genomic analysis and effectiveness in preventing pneumonia
Source: Appl Microbiol Biotechnol. 2024 Jul 27;108:428. doi: 10.1007/s00253-024-13208-0 (PMC11283397; doi:10.1007/s00253-024-13208-0)
Supplement: Supplementary file 1 [file 253_2024_13208_MOESM1_ESM.pdf]

Applied Microbiology and Biotechnology

A novel *Saclayvirus Acinetobacter baumannii* phage genomic analysis and effectiveness in preventing pneumonia

Shibin Li[1], Bingdong Wei[2], Le Xu[1], Cong Cong[1], Bilal Murtaza[1], Lili Wang[1], Xiaoyu Li[1], Jibin Li[4], Mu Xu[5], Jiajun Yin[3*], Yongping Xu[1,5*]

[1]School of Bioengineering, Dalian University of Technology, Dalian 116024, China.

[2]Institute of Animal Nutrition and Feed Science, Jilin Academy of Agricultural Sciences, Gongzhuling 136100, China.

[3]Department of General Surgery, Affiliated Zhongshan Hospital of Dalian University, Dalian 116300, Liaoning, P. R. China

[4]Liaoning Innovation Center for Phage Application Professional Technology, Dalian 116620, China

[5]Dalian SEM Bio-Engineering Technology Co. Ltd., Dalian 116620, China.

corresponding author:

Yongping Xu

Address: School of Bioengineering, Dalian University of Technology, Dalian, China.

Email: xyping@dlut.edu.cn

TEL: 0411-84709800

FAX:86-411-84707983

Jiajun Yin

Address: Department of General Surgery, Affiliated Zhongshan Hospital of Dalian University, Dalian, China.

Email: yinjiajun@dlu.edu.cn

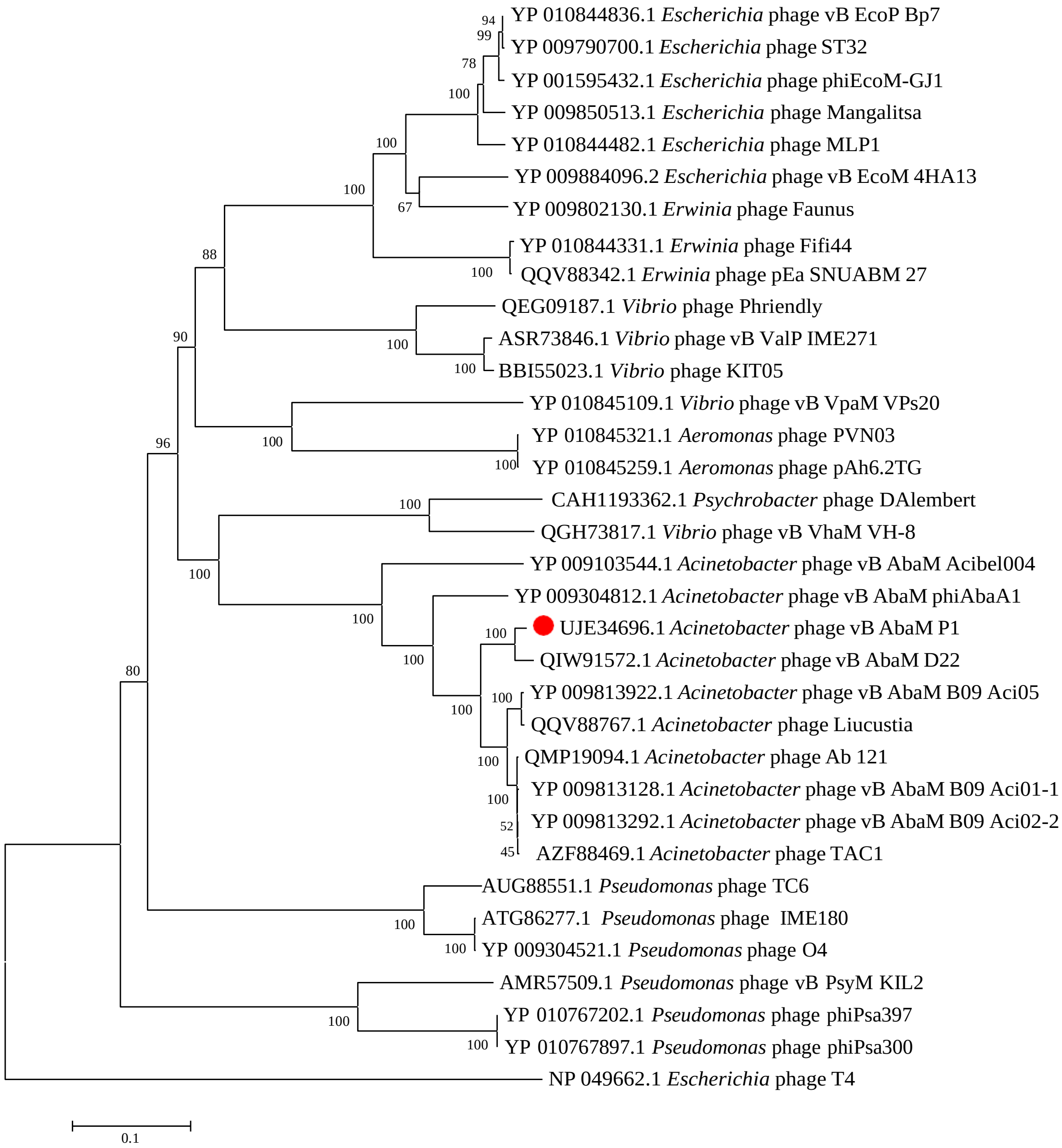

Fig. S1 A phylogenetic tree constructed with the DNA polymerase protein of phage vB_AbaM_P1. Comparison with corresponding proteins from other phages in the NCBI protein database were performed by BLASTp search (default parameters, but maximum target sequence set to 250). The tree was generated using the MEGA7 tool with the neighbor-joining method and 1000 bootstrap replicates. The Escherichia virus T4 protein was used as an outgroup to root the tree

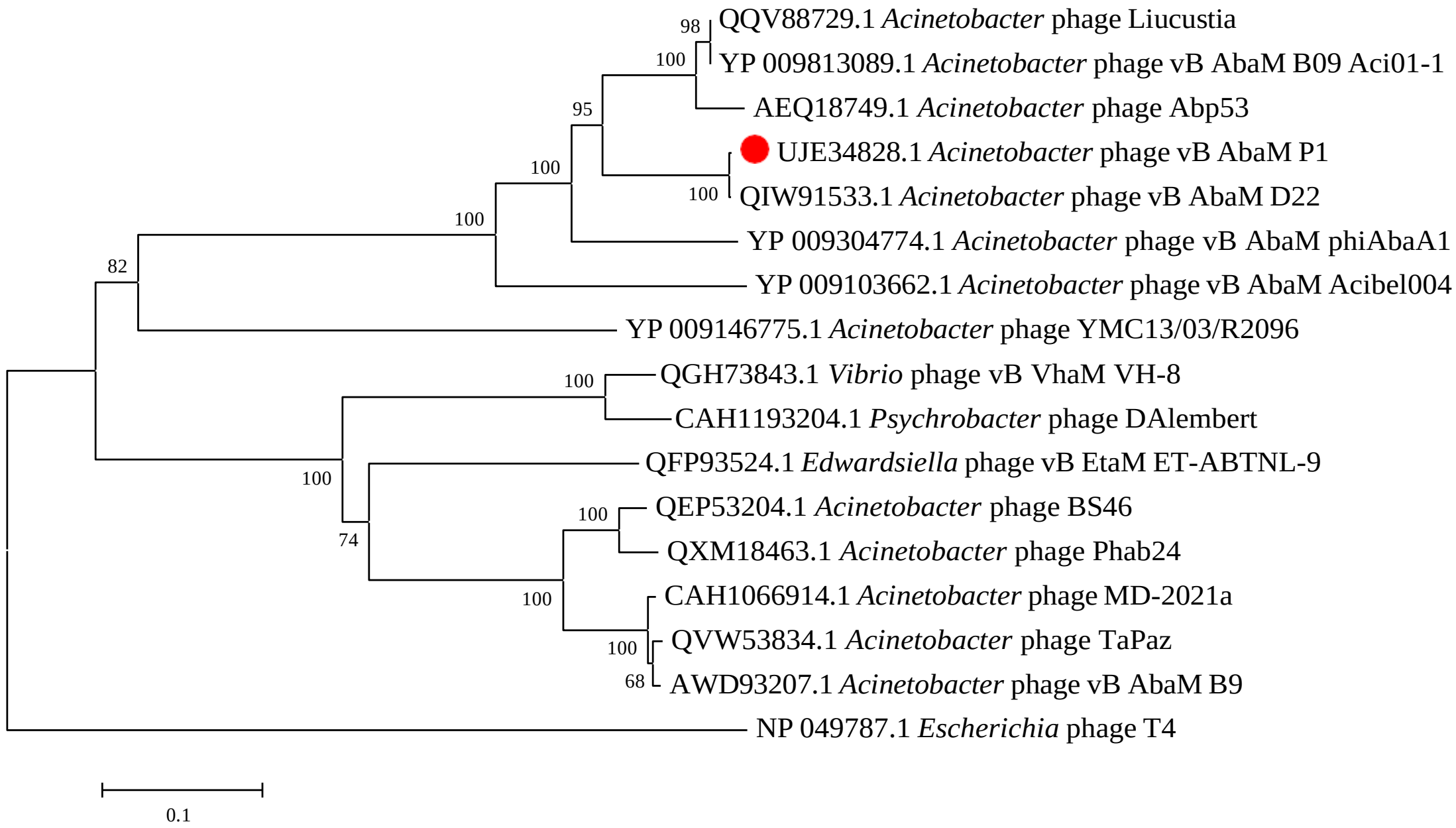

Fig. S2 A phylogenetic tree constructed with the major capsid protein of phage vB_AbaM_P1. Comparison with corresponding proteins from other phages in the NCBI protein database were performed by BLASTp search (default parameters, but maximum target sequence set to 250). The tree was generated using the MEGA7 tool with the neighbor-joining method and 1000 bootstrap replicates. The Escherichia virus T4 protein was used as an outgroup to root the tree

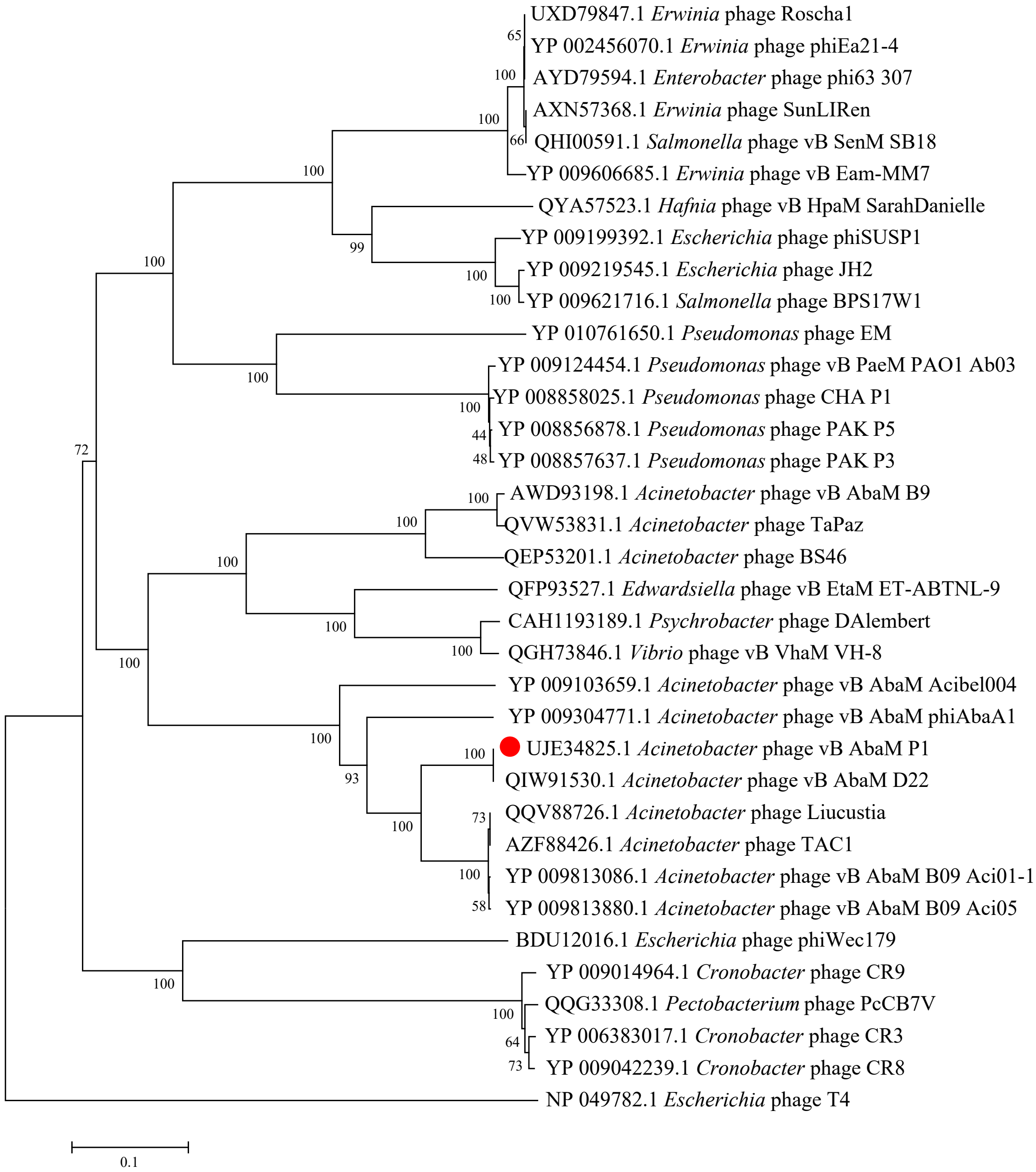

Fig. S3 A phylogenetic tree constructed with the portal protein of phage vB_AbaM_P1. Comparison with corresponding proteins from other phages in the NCBI protein database were performed by BLASTp search (default parameters, but maximum target sequence set to 250). The tree was generated using the MEGA7 tool with the neighbor-joining method and 1000 bootstrap replicates. The Escherichia virus T4 protein was used as an outgroup to root the tree

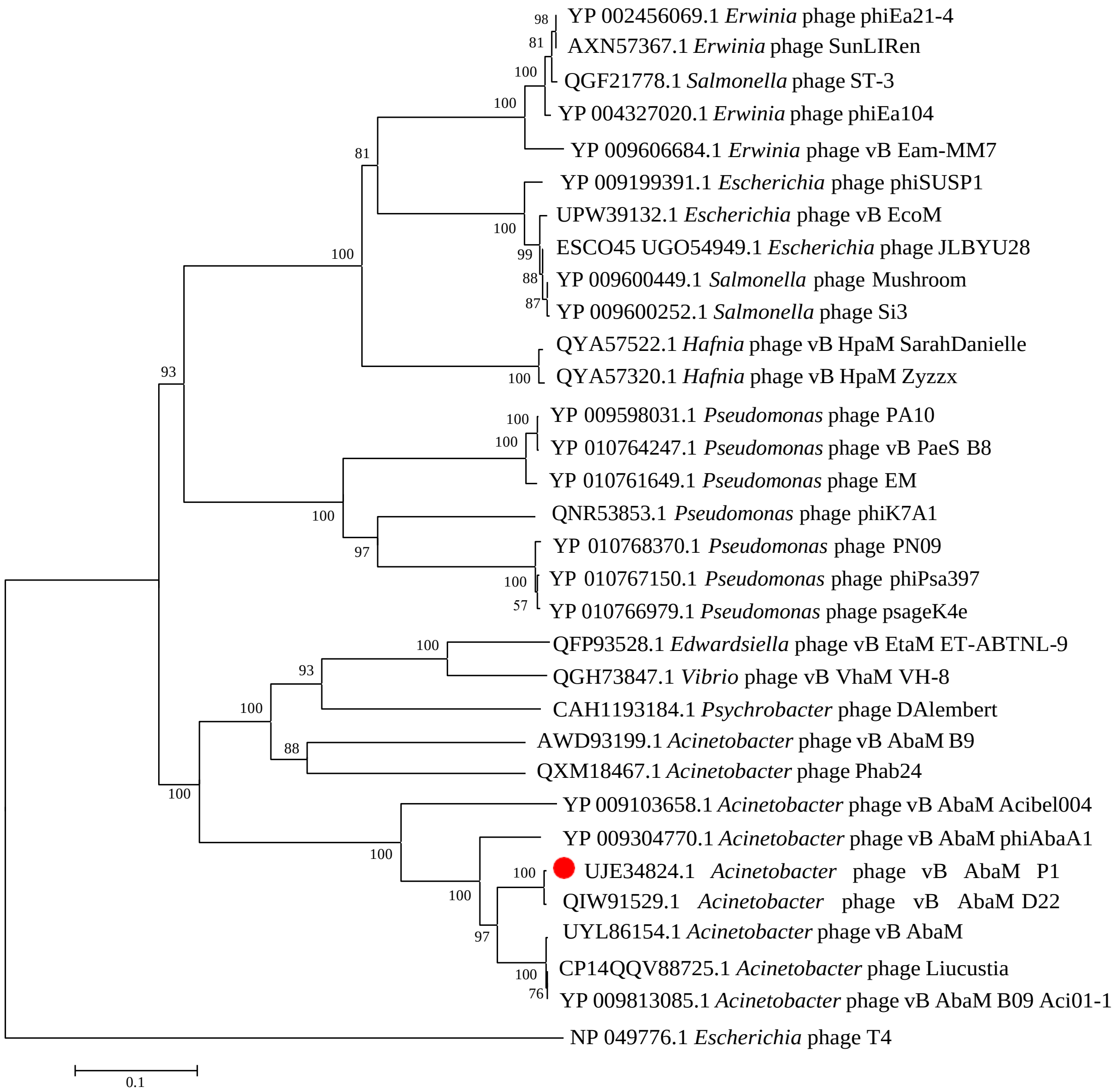

Fig. S4 A phylogenetic tree constructed with the terminase large subunit of phage vB_AbaM_P1. Comparison with corresponding proteins from other phages in the NCBI protein database were performed by BLASTp search (default parameters, but maximum target sequence set to 250). The tree was generated using the MEGA7 tool with the neighbor-joining method and 1000 bootstrap replicates. The Escherichia virus T4 protein was used as an outgroup to root the tree
